# Chemical Composition, Antifungal and Anti-Biofilm Activities of Volatile Fractions of *Convolvulus althaeoides* L. Roots from Tunisia

**DOI:** 10.3390/molecules27206834

**Published:** 2022-10-12

**Authors:** Soukaina Hrichi, Raja Chaâbane-Banaoues, Filippo Alibrando, Ammar B. Altemimi, Oussama Babba, Yassine Oulad El Majdoub, Habib Nasri, Luigi Mondello, Hamouda Babba, Zine Mighri, Francesco Cacciola

**Affiliations:** 1 Laboratory of Physico-Chemistry of Materials, Faculty of Sciences of Monastir, University of Monastir, Monastir 5000, Tunisia; 2 Laboratory of Parasitology and Mycology (LP3M), Department of Clinical Biology, Faculty of Pharmacy of Monastir, University of Monastir, Monastir 5000, Tunisia; 3 Chromaleont s.r.l., c/o Department of Chemical, Biological, Pharmaceutical and Environmental Sciences, University of Messina, 98122 Messina, Italy; 4 Department of Food Science, College of Agriculture, University of Basrah, Basrah 61004, Iraq; 5 College of Medicine, University of Warith Al-Anbiyaa, Karbala 56001, Iraq; 6 Department of Chemical, Biological, Pharmaceutical and Environmental Sciences, University of Messina, 98122 Messina, Italy; 7 Department of Sciences and Technologies for Human and Environment, University Campus Bio-Medico of Rome, 00128 Rome, Italy; 8 Department of Biomedical, Dental, Morphological and Functional Imaging Sciences, University of Messina, 98122 Messina, Italy

**Keywords:** *Convolvulus althaeoides* L. roots, gas chromatography, mass spectrometry, volatile fractions, antifungal, *Candida* spp. biofilm

## Abstract

The antifungal drugs currently available and mostly used for the treatment of candidiasis exhibit the phenomena of toxicity and increasing resistance. In this context, plant materials might represent promising sources of antifungal agents. The aim of this study is to evaluate for the first time the chemical content of the volatile fractions (VFs) along with the antifungal and anti-biofilm of *Convolvulus althaeoides* L. roots. The chemical composition was determined by gas chromatography coupled to a flame ionization detector and mass spectrometry. In total, 73 and 86 chemical compounds were detected in the n-hexane (VF1) and chloroform (VF2) fractions, respectively. Analysis revealed the presence of four main compounds: *n*-hexadecenoic acid (29.77%), 4-vinyl guaiacol (12.2%), bis(2-ethylhexyl)-adipate (9.69%) and eicosane (3.98%) in the VF extracted by hexane (VF1). *n*-hexadecenoic acid (34.04%), benzyl alcohol (7.86%) and linoleic acid (7.30%) were the main compounds found in the VF extracted with chloroform (VF2). The antifungal minimum inhibitory concentrations (MICs) of the obtained fractions against *Candida albicans*, *Candida glabrata* and *Candida tropicalis* were determined by the micro-dilution technique and values against *Candida* spp. ranged from 0.87 to 3.5 mg/mL. The biofilm inhibitory concentrations (IBF) and sustained inhibition (BSI) assays on *C. albicans*, *C. glabrata* and *C. tropicalis* were also investigated. The VFs inhibited biofilm formation up to 0.87 mg/mL for *C. albicans*, up to 1.75 mg/mL against *C. glabrata* and up to 0.87 mg/mL against *C. tropicalis*. The obtained results highlighted the synergistic mechanism of the detected molecules in the prevention of candidosic biofilm formation.

## 1. Introduction

The plant world has always been considered as a source of many natural compounds, which has prompted researchers to study many plant species. The new findings have demonstrated that plants are enriched with many bioactive secondary metabolites such as terpenoids, fatty acids, phenolics and alkaloids [1,2], which are characterized by high antifungal proprieties [3,4].

*Convolvulus* is a genus of the family Convolvulaceae which is found in all Mediterranean regions. This genus includes about 250 species such as *Convolvulus prostrates, Convolvulus austroaegyptiacus, Convolvulus pilosellifolius* Desr., *Convolvulus pluricaulis* and *Convolvulus arvensis* L. [5], which have been reported to possess medicinal effects and in particular antioxidant, anti-inflammatory, antimicrobial and anticancer properties [5,6,7,8]. In Tunisia, *Convolvulus althaeoides* L. has been the most characterized species in terms of chemical composition, and previous studies demonstrated its fruitful content of its extracts in terms of flavonoids, phenol terpenes, carotenoids, polyphenols, chorophylls, and carotenoids [9,10]. Moreover, oxygenated monoterpenes, oxygenated sesquiterpenes and sesquiterpene hydrocarbons, determined by gas chromatography, have been reported as constituens of the flowers of such a species [11]. A new acylated anthocyanintrioside was isolated by extraction with methanol solution from flowers of *C. althaeoides* L. from Portugal and identified from MALDI-TOF and NMR spectrophotometric technologies [12]. Our previous study revealed that ethanolic extract of *C. althaeoides* L. leaves were rich in antioxidant polyphenols, while dichloromethane extract showed the highest rate of carotenoids [8]. Ethyl acetate and ethanol extracts were the most active against dermatophytes (*T. rubrum, T. menthagrophytes* and *M. canis*), with inhibition percentages reaching 100% at the concentration of 50 mg/mL. Furthermore, ethyl acetate and ethanolic extracts showed maximum inhibition potential with minimum inhibitory fungicidal concentrations (MCFs) ranging from 0.78 to 6.25 mg/mL when tested on *Candida* spp. cultures. Moreover, extracts of winter leaves of the *C. athaeoides* L. showed inhibitory effects up to 90% of *Candida albicans* germ tube formation, at a concentration of 3.1 mg mL^−1^.

The genus *Candida* consists of more than 200 species, and only some of them are pathogenic to humans. These are yeast fungi, and the best-known species are represented by *C. albicans, C. tropicalis, C. glabrata* and *C. krusei*, generally associated with pathological conditions [13].

*Candida* spp. are part of natural microbiota of immunocompetent individuals, but in case of health weakness, *Candida* isolates can become opportunist and cause major common hospital-acquired systemic infections with high mortality rates [14].

According to the US Center of Disease Control and Prevention (CDC) in 2019, a report on the antibiotic resistance threat reveled more than 34,000 cases and 1700 deaths annually were due to drug-resistant *Candida* spp. In addition, an emerging multidrug resistance in *Candida* spp. infections has been reported, making them difficult to treat. In fact, fluconazole resistance has been demonstrated in 7% of *Candida* sepsis and *C. glabrata* resistance to Echinocandine was constantly reported over the past two decades [15].

The capacity of pathogenic species of the *Candida* genus to infect and harm is highly related to its virulence factors including adhesion, phenotypic switching germ tube formation, dimorphism, production of hydrolytic enzymes and biofilm formation [16]. The formation of *Candida* biofilms has a significant clinical impact due to their increased resistance to antifungal treatments and the ability of biofilms to resist immune mechanisms [17].

For many years, a range of different synthetic chemicals (aromatic hydrocarbons, amphotericin B, benzimidazoles and sterol) have been used as antifungal agents to inhibit the growth of plant pathogenic fungi [18]. Plant components with antifungal properties are considered as a valuable resource in the treatment of fungal diseases. There is a growing interest in the use of natural products such as essential oils, which are volatile and non-volatile natural compounds comprising a complex mixture of terpenoids, alcohol compounds, phenylpropanoids, aldehydes and acidic compounds, among others [19]. Due to their strong odor, they have been traditionally used as natural flavorings and recently as natural antimicrobials.

The essential oil composition of the roots of *C. althaeoides* L. and its potential biomedical value have not been scientifically investigated. Therefore, the present study aims to investigate for the first time the volatile profile and the antifungal and anti-biofilm properties of the essential oil of the Tunisian cultivar of *C. althaeoides* L., as well as to elucidate the role and the relative importance of their dominant constituents to ensure the maintenance of biological activity.

## 2. Results and Discussion

### 2.1. Yields and Chemical Composition of the VFs

**Figure 1:**
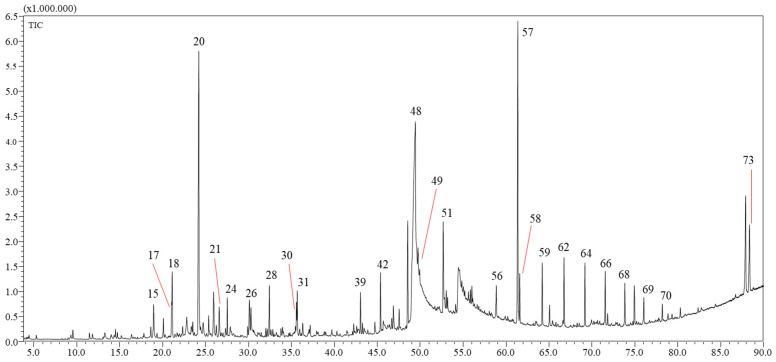
GC-MS profile of the hexane fraction (VF1) of *C. althaeoides* L. roots.

**Figure 2:**
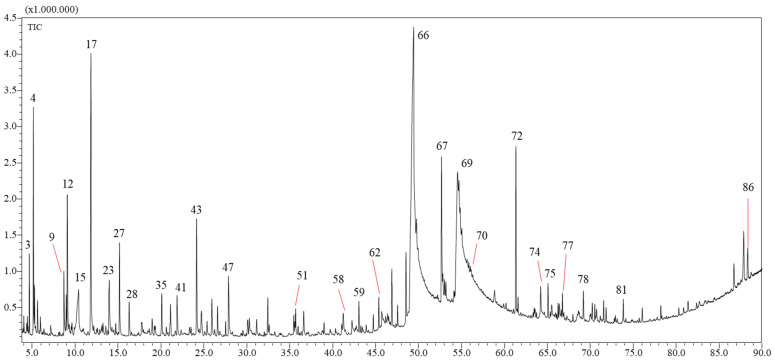
GC-MS profile of the chloroform fraction (VF2) of *C. althaeoides* L. roots.

The *n*-hexane (VF1) and chloroform (VF2) VFs were obtained in yields of 0.012 ± 0.005% and 0.009 ± 0.003 (*w*/*w*) of the dry mass of *C. althaoides* L. roots. This is comparable to the yield of the essential oil obtained from the flowers of this species 0.007% [11]. Chemical profiles of root *C. althaeoides* L. volatiles fractions were determined by GC-MS, and results are presented in Table 1 and Table 2, in Tables S1 and S2 and in Figure 1 and Figure 2. Seventy-three compounds (Table 1 and Table S1) and eighty-six compounds (Table 2 and Table S2) were identified from VF1 and VF2, respectively.

The content of VF1, achieved by the GC-MS technique, was characterized as fatty acids (33.50%), alcohols (17.83%), alkanes (15.33%), esters (14.54%), alkenes (8.73%), oxygenated monoterpenes (3.85%), carboxylic acids (2.71%), apocarotenes (1.29%), ketones (0.84%), aldehyde (0.57%), sesquiterpene hydrocarbons (0.42%) and triterpenes (0.39%). The most abundant components are: *n*-hexadecanoic acid (29.77%) among fatty acids; 4-vinylguaiacol (12.2%) and octadecanol (2.89%); eicosane (2.11%) among alkanes; bis-(2-ethylhexyl)adipate (9.69%) and diisobutyl phthalate (1.55%) among the esters; eicosene (3.98%) among the alkenes; carvone (1.77%) among the oxygenated monoterpenes; benzenepropanoic acid, 3,5-bis(1,1-dimethylthyl)-4-hydroxy-, octadecyl ester (2.71%) among carboxylic acids; 7,9-di-tert-butyl-1-oxaspiro(4,5)deca-6,9-diene-2,8-dione (0.65%) among apocarotenes; 5-dodecyldihydro-2(3*H*)-furanone (0.52%) among ketones; *n*-nonanal (0.14%) among aldehydes; pentadecylic acid (0.42%) among sesquiterpene hydrocarbons; squalene (0.28%) among triterpenes and cadalene (0.11%) among sesquiterpene hydrocarbons.

The content of VF2, achieved by the GC-MS technique, was characterized by fatty acids (41.74%), alcohols (21.01%), alkanes (8.51%), esters (7.65%), aldehydes (6.51%), pyridine (5.06%), carboxylic acids (2.86%), oxygenated monoterpenes (2.16%), alkenes (1.55%), ketones (1.17%), phenylpropanoids (0.64%), oxygenated sesquiterpenes (0.46%), triterpenes (0.34%), apocarotenes (0.17%) and sesquiterpene hydrocarbons (0.09%). The most abundant components were *n*-hexadecanoic acid (34.04%) and linoleic acid (7.30%) among the fatty acids; benzyl alcohol (7.86%) and octadecanol (3.53%) among the alcohols; docosan (2.43%) among alkanes; bis-(2-ethylhexyl)adipate (3.57%) among esters; furfural (3.24%) and vanillin (1.49%) among aldehydes; 3-ethenyl-pyridine (2.43%) and beta-lutidine (1.29%) among pyridine; *n*-hexanoic acid (1.91%) among carboxylic acids; myrtenol (0.46%) among oxygenated monoterpenes; octadec-1-ene (0.67%) among alkenes; 5-dodecyldihydro-2(*3H*)-furanone (0.52%) among ketones; (*E*)-coniferyl alcohol (0.67%) among phenylpropanoids; pentadecylic acid (0.36%) among oxygenated sesquiterpenes; squalene (0.34%) among triterpenes; benzophenone (0.1%) among apocarotenes and 1,6-dimethyl-4-(1-methylethyl)-naphthalene (0.09%) among sesquiterpene hydrocarbons.

*n*-Hexadecanoic (palmitic) acid (C_16_H_32_O_2_) was the most abundant compound, which has several biological activities: antimicrobial, antieczematous, antiseborrheic, sclerosing, antihypoxic, antimutagenic, fibrinolytic, anti-inflammatory, antisecretory, cytoprotective and anesthetic [20]. In fact, *n*-hexadecanoic acid is one of the primary metabolites produced during microbial degradation because of the oxidized end of the molecule is used as the initial site for the β-oxidation process, whereas microbial oxidation of saturated hydrocarbons (*n*-hexadecane) cannot be initiated as easily as for *n*-hexadecanoic acid [21]. 4-vinyl guaiacol is a vinyl phenol produced by the decarboxylation of ferulic acids. Its phenolic structure allows it to play the role of a natural antioxidant. Indeed, the position of the methoxy group on the electron-donating phenolic ring is also a factor that increases the stability of the phenoxy radical and thus its antioxidant effectiveness [22].

The chemical composition of the VFs of the roots of *C. althaeoides* L. are herein reported for the first time. Only one study on the essential oil of the flower of *C. althaeoides* L. grown in Tunisia has been carried out [11], which reveled 24 compounds, representing 95.50% of the total compositions. The oil was characterized by a high proportion of sesquiterpene hydrocarbons (36.30%), followed by oxygenated sesquiterpenes (34.70%) and oxygenated monoterpenes (24.50%). The main compounds are germacrene D (12.50%), T-cadinol (11.80%) and verbenone (6.90%).

### 2.2. Antifungal Activity

**Figure 3:**
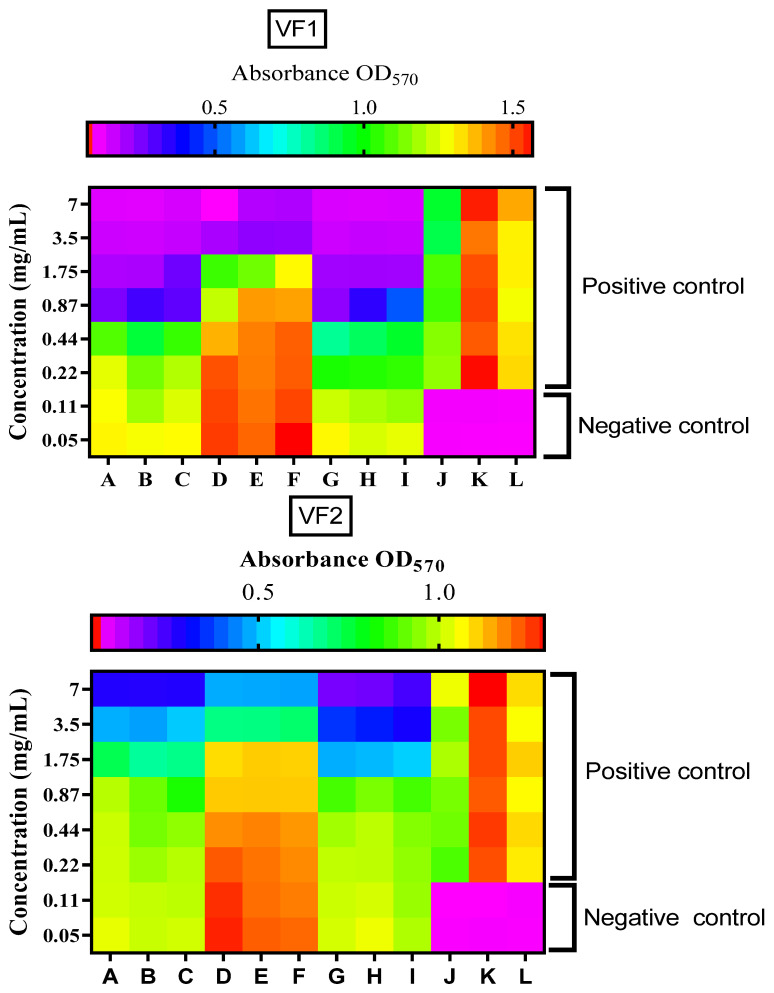
Heatmaps representing spectrophotometric reads (OD) of the microdilution plates at 570 nm using the Multiskan™ FC Microplate Photometer after susceptibility test on *Candida* spp. to VF1 and VF2 fractions.

The outputs of the microdilution test of VF1 and VF2 from *C. althaeoides* L. roots against *Candida* spp. at different concentrations, read with Multiskan™ FC Microplate Photometer (Thermo Scientific, Waltham, MA, USA), are represented as heatmaps (Figure 3). These illustrations demonstrated a good level of reproducibility in our repeats and a difference of inhibition for both VF1 and VF2 fractions against each fungal strain. *C. albicans* and *C. tropicalis* demonstrated the same susceptibility level to VF1 and VF2 fractions with MICs at 0.87 and 1.75 mg/mL, respectively. The MICs for the Amphotericin B were 0.125 × 10^−3^ mg/mL, 0. 062 × 10^−3^ mg/mL and 0.250 × 10^−3^ mg/mL for *C. albicans*, *C. glabrata* and *C. tropicalis*, respectively (data not shown). The MFCs against these strains were reported at 1.75 mg/mL for both VF1 and VF2 fractions of *C. albicans* and *C. tropicalis*. However, *C. glabrata* seems to be less susceptible to VF1 and VF2 fractions with MICs/MFCs at 3.5 mg/mL and 7 mg/mL, respectively. In agreement with the photochemical results of the two VFs, the diversity and the richness of the two VFs in volatile compounds can explain their antifungal potential. Indeed, the main active compounds of the VFs of *C. althaeoides* L. roots were *n*-hexadecenoic acid [23], bis (2-ethylhexyl) adipate [24], benzyl alcohol [25] and linoleic acid [26] which are known as antifungal agents. In fact, it was reported previously that the *n*-hexadecanoic acid or palmitic acid, a saturated fatty acid, abundant compound in VF1 and VF2, showed antifungal activities against mycelial growth and spore production reaching 37.20% and 71.70% by 2 mM concentration [27], respectively. Eicosane (C_20_H_42_), a straight chain alkane composed of 20 carbon atoms [28]; carvone (C_10_H_14_O), a *p*-menthane monoterpenoid that consists of cyclohex-2-enone having methyl and isopropenyl substituents at positions 2 and 5, respectively [29], vanillin (C_8_H_8_O_3_) a phenolic aldehyde [30] and guaiacol (C_7_H_8_O_2_), a monomethoxybenzene that consists of phenol with a methoxy substituent at the ortho position [31], were proven to have high antifungal potential at a limited concentration.

Furthermore, it has been demonstrated by several studies that extracts, VFs and essential oils extracted from plants of the *Convolvulus* genus strongly inhibit the growth of various fungal pathogens [7,9,10,11]. A previous study showed an inhibitory effect of up to 0.31 ± 0.10 mg/mL for *C. althaeoides* oil against *Pseudomonas aeruginosa* and *Enterococcus faecalis* [11].

Columns A, B and C: testing the susceptibility of *C. albicans* to VFs different concentrations (0.05–7 mg/mL). Columns D, E and F: testing the susceptibility of *C. glabrata* to different concentrations (0.05–7 mg/mL). Columns G, H and I: testing the susceptibility of *C. tropicalis* to VFs different concentrations (0.05–7 mg/mL). J, K and L: upper *C. albicans*, *C. glabrata* and *C. tropicalis* positive control wells, respectively; down: negative control wells (RPMI 1640-2% glucose).

### 2.3. Anti-Biofilm Activity

#### 2.3.1. Inhibition of Biofilm Formation (IBF)

**Figure 4:**
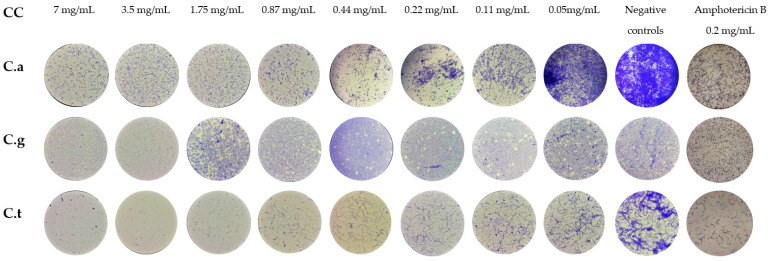
Micrographs of *Candida* spp. cells treated with various concentrations of VF1 for Inhibition of Biofilm Formation (IBF). CC: Concentration, C.a: *Candida albicans*, C.g: *Candida glabrata*. C.t: *Candida tropicalis.*

**Figure 5:**
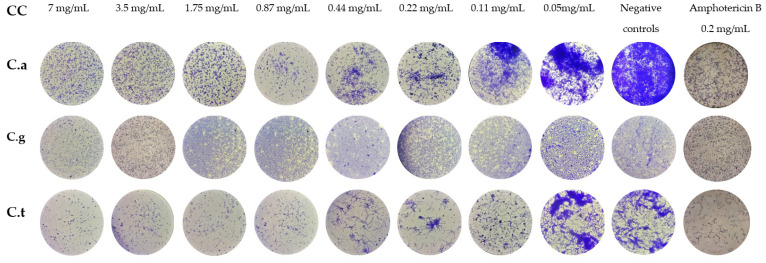
Micrographs of *Candida* spp. cells treated with various concentrations of VF2 for Inhibition of Biofilm Formation (IBF). CC: Concentration, C.a: *Candida albicans*, C.g: *Candida glabrata*. C.t: *Candida tropicalis.*

Microscopic observation of biofilm plates permitted us to evaluate the IBF potential of VF1 and VF2 at different concentrations on *Candida* spp. (Figure 4 and Figure 5). Compared to the positive controls of growth; both VFs demonstrated a high inhibition potential on *Candida* Biofilm formation. In fact, VF1 and VF2 induced 80% of reduction of *C. albicans* biofilm biomass up to 0.21 mg/mL and 0.43 mg/mL, respectively. The filamentation was inhibited by both of VFs at concentration of 1.75 mg/mL. *Thymus capitatus* (0.12 mg/mL) and *Cinnamomum verum* (0.62 mg/mL) essential oils were efficient against *C. albicans* biofilm formation at a similar level with 80.60% and 85.57% of inhibition, respectively. Moreover, micrographs of biofilm formed after 24 h incubation demonstrated a filamentation inhibition at half MICs for both of them [32]. *Candida albicans* biofilms are inherently resistant to antifungal drugs, mainly azoles and polyenes [33]. Thus, essential oils can offer new perspectives as an alternative treatment.

A higher potential of IBFs has been observed on *C. tropicalis* for both VF1 and VF2 fractions with biomass inhibitions of 100% and 90% up to 3.5 mg/mL and 0.87 mg/mL, respectively. A total filamentation inhibition on *C. tropicalis* was observed for VF1 and VF2 at 0.87 mg/mL and 1.5 mg/mL, respectively. *C. tropicalis* have been demonstrated sensitive to treatment with essential oils of *Thymbra capitata* (0.62 µL/ml) with percentages of reduction of biofilm biomass and reduction of biofilm metabolism of 67.82% and 97.47%, respectively [34]. Essential oils from *Pelargonium graveolens* have been described to possess a high continence in geraniol and linalool, which are α-terpineol present in both VF1 and VF2 fractions from this study and were 90% inhibitory to *C. tropicalis* biofilm formation at 8 mg/mL and reduced only 25% of the biofilm at a concentration similar to our effective concentration (0.87 mg/mL).

Both VF1 and VF2 demonstrated a reduced inhibition potential with a reduction of 50% of *C. glabrata* biofilm biomass up to 3.5 mg/mL concentration. With the exception of *Candida* spp., *Candida glabrata* biofilms were more resistant as the reduction in biomass by both VF1 and VF2 remained below 60%. This limited effect can be explained by the particularity of this yeast, which possesses a structure in different multilayers making its biofilm more compact than other *Candida* strains [35].

#### 2.3.2. Biofilm Sustained Inhibition (BSI)

**Figure 6:**
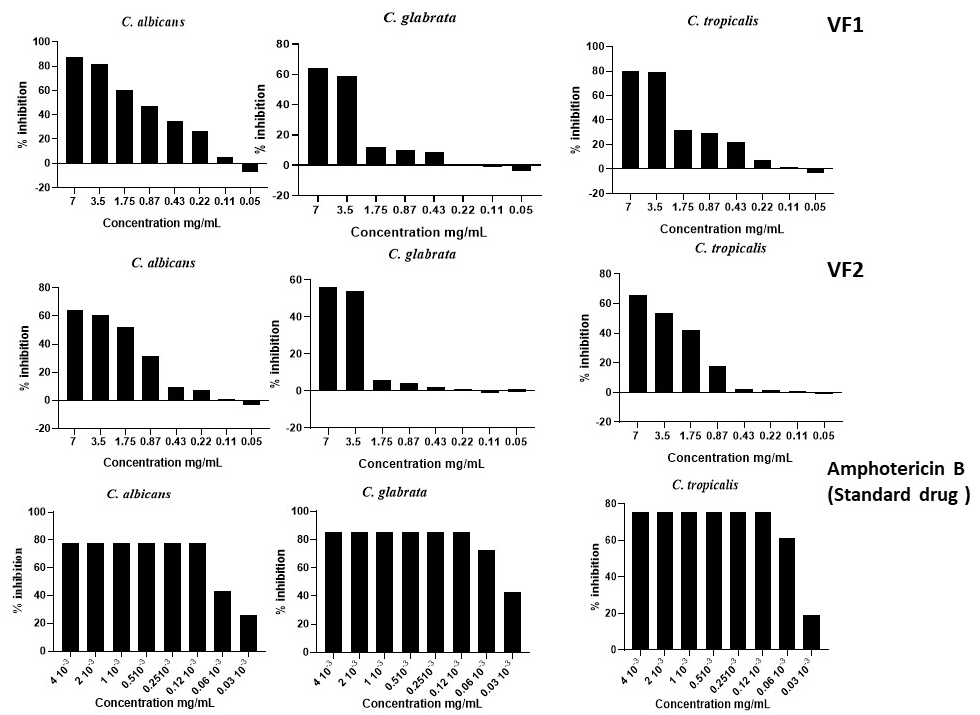
Effect of different concentrations of VFs on the biofilm of *Candida* spp. (BSI).

The evaluation of the effect of the two VFs of *C. althaeoides* L. roots on the biofilm formation of *C. albicans, C. glabrata* and *C. tropicalis* demonstrated that both of them had an inhibitory potential on biofilm formation (Figure 6). One-way ANOVA computed a *p*-value < 0.05 (significant effect of different treatments) as percentages of the untreated control. At a concentration of 7 mg/mL, the inhibition percentage of VF1 on *C. albicans* biofilm formation was 87.23%, while the inhibition rate of VF2 was 65.91%. VF1 and VF2 showed 63.98% and 56.06% inhibition on the BSI of *C. glabrata* at a concentration of 7 mg/mL, respectively (Figure 4). Similar yields were reported against the biofilm formation of the *C. tropicalis* strain, which was inhibited by 79.74% and 65.91% for VF1 and VF2, respectively. The BSI on *Candida* spp. can be explained by several factors. Indeed, inhibition of adhesion is the first significant step in preventing biofilm formation. The anti-biofilm capability of the two VFs with a preference for VF1 can be attributed to its ability to inhibit the attachment of *Candida* spp. to surfaces. For pathogens, biofilm plays a key role in nullifying the effect of antifungal and anti-biofilm agents. The BSI is the key step in reducing the pathogenic effect of *Candida* spp. The anti-biofilm effect of these fractions can be attributed to specific components. The two VFs tested in this study have been demonstrated to harbor a wide range of phytochemical components that could be considered responsible for a more or less biological effect. The activity of these tested VFs varied according to its formulation. Indeed, the high amounts of fatty acids and terpenoids were attributed to a better anti-biofilm activity [36,37]. *n*-Hexadecanoic acid (C_16_H_32_O_2_), namely, palmitic acid, a saturated long-chain fatty acid with a 16-carbon backbone [23]; cuminaldehyde (C_10_H_12_O), a benzaldehyde substituted by an isopropyl group at position 4 [38]; linoleic acid (C_18_H_32_O_2_), an octadecadienoic acid in which the two double bonds are at positions 9 and 12 and have *Z* (cis) stereochemistry [39]; and benzyl alcohol (C_7_H_8_O), an aromatic alcohol that consists of benzene bearing a single hydroxymethyl substituent [25] were the main compounds present in these fractions that were associated with anti-biofilm activities. The synergistic effects of such compounds might be responsible for the observed anti-biofilm activity [40]. The antimicrobial activity of these compounds is based on different mode of action, as described for cuminaldehyde, which has the propriety to disrupt *Staphylococcus aureus* membranes and bind to DNA through the groove to impact normal cellular function [41]. Benzyl Alcohol has been proven to induce attack on bacterial membranes and protein denaturation, following an increase of reactive oxygen species levels [42]. Linoleic acid as fatty acid was involved in affecting membrane permeability (pore formation and membrane destabilization), reducing hydrophobicity but being able to interfere with microbial metabolism or signaling [43]. Palmitic acid was observed to enhance hyphal growth for *C. tropicalis* through upregulation of hyphal wall protein (HWP1) and enhanced filamentous growth (EFG1) genes [44].

## 3. Materials and Methods

### 3.1. Plant Material

The roots of *C. althaeoides* L. were collected in the region of Kondar (TUNISIA) in January 2017, a rural area of the Tunisian Sahel located about 30 km north-west of the governorate of Sousse. The plant material was naturally dried in the shade at room temperature for 2–3 weeks, ground into a fine powder and later used for extractions.

### 3.2. Chemicals and Reagents

All solvents used in the experiments (hexane and chloroform) were analytical grade (Merck Life Science, Merck KGaA, Darmstadt, Germany). Dimethyl sulfoxide (DMSO) was purchased from BIO BASIC INC (Desk, Markham, ON, Canada). Culture media were purchased from Sigma-Aldrich (CHEMIE GmBH, Riedstr, Germany). RPMI-1640 medium was purchased from Gibco and stored at 4 °C. Glucose solution 30% (Siphal, Tunisia).

### 3.3. Preparation of VF from Roots of C. althaeoides L.

The whole process from preparing VFs from root powder was followed according to the method described by Hrichi et al. [40]. Briefly, 100 g of root powder was subjected to hydrodistillation for 3 h. The collected hydrodistillates were subjected to a liquid–liquid extraction with hexane and chloroform, successively. The two VFs, VF1 (hexane) and VF2 (chloroform), were dried over anhydrous sodium sulphate (Na_2_SO_4_) and stored in a refrigerator at −4 °C until analysis. The yield of VFs was calculated following the next formula:

% Yield of VFs = [Weight of VFs/Weight of dried roots] × 100%

### 3.4. Gas Chromatography–Mass Spectrometry (GC–MS) Analysis

The composition of VFs of *C. althaeoides* L. was identified by GC-MS analysis. GC-MS analysis was carried out on a GC–MS-QP2020 system (Shimadzu, Kyoto, Japan) equipped with an “AOC-20i” system auto-injector, and separation was attained in a column SLB-5ms (30 m in length × 0.25 mm in diameter × 0.25 µm in thickness of film, Merck Life Science, Merck KGaA, Darmstadt, Germany). The temperature of the injection port was set at 50 °C and afterwards increased up to 350 °C (increase rate: 3 °C/min; holding time: 5 min). The GC parameters were as follows: injection temperature, 280 °C; injection volume, 1.0 µL (split ratio: 10:1); pure helium gas, 99.9%; linear velocity, 30.0 cm/s; inlet pressure, 26.7 KPa. The MS conditions included an interface temperature of 220 °C, a source temperature of 250 °C and a mass scan range of 40–660 amu. The peak of the samples was identified by using the “FFNSC 3.01” (Shimadzu Europa GmbH, Duisburg, Germany) and “W11N17” (Wiley11-Nist17, Wiley, Hoboken, NJ, USA; Mass Finder 3). Each compound was identified applying a MS similarity match and an LRI filter. Linear retention indices (LRI) were calculated by using a C7-C40 saturated alkanes reference mixture (49452-U, Merck Life Science, Merck KGaA, Darmstadt, Germany). A relative quantity on the basis of peak area percentages was carried out.

### 3.5. Antifungal Activity

#### 3.5.1. Fungal Strains

Reference strains of *Candida* spp. used in this study belonged to the American Type Culture Collection (*Candida albicans* ATCC 90028, *Candida tropicalis* ATCC 66029 and *Candida glabrata* ATCC 64677). These fungal strains were supplied by the laboratory of Parasitology-Mycology of the Fattouma Bourguiba teaching hospital of Monastir. Yeast strains were maintained on Sabouraud-Chloramphenicol Agar (SCA).

#### 3.5.2. Determination of the Minimum Inhibitory Concentrations (MICs)

MICs of the VFs were determined using the microdilution technique, as previously described by Hrichi et al. [10], with some modification. Briefly, microtiter plates with 96 flat-bottomed wells were used, and serial dilutions of the test substance (7.00, 3.50, 1.75, 0.87, 0.44, 0.22, 0.11 and 0.05 mg/mL) were prepared using RPMI-1640 media supplemented with 2% glucose. *Candida* spp. strains were subjected to a susceptibility test face to Amphotericin B concentrations ranging from 16 × 10^−3^ mg/mL to 0.03 × 10^−3^ mg/mL as drug standard. Inoculums for the assay were prepared for each strain using diluting a 24 h fresh colony from sabouraud chloramphenicol agar into RPMI-1640-2% glucose and adjusting it to 2.5–5 × 10^5^ CFU mL^−1^. The final concentration was confirmed using a Malassez counting chamber. Working inoculums (100 µL) were added to the 96-well plates, which were incubated at 37 °C for 24 h. A fungal suspension in the medium was used as growth positive control, and non-inoculated medium RPMI-G 2% (200 µL) was included as a negative control. All experiments were repeated in triplicate. MICs were determined by spectrophotometic lecture at 570 nm to control the growth of fungal strains and defined as the lowest concentration of the VFs produced growth inhibition compared with the growth in the untreated control well.

#### 3.5.3. Minimum fungicidal concentrations (MFCs)

The MFCs were determined using a subculture of 10 μL from MICs wells onto sabouraud chloramphenicol agar plates. The plates were incubated at 37 °C for 24 h. The concentration that induces no visible colony growth after subsequent 24 h incubation was accepted as MFC, which is the lowest concentration without visible biomass growth and corresponding to the death of 99.90% of the original inoculum.

### 3.6. Inhibition of Biofilm Formation (IBF)

The VFs VF1 and VF2 were tested for their capacity to inhibit *Candida* spp. biofilm formation. Serial dilutions of (from 7 to 5 × 10^−2^ mg/mL) were incubated with 5–2.5 × 10^5^ CFU mL^−1^ fungal cells in RPMI 1640-2% glucose. Inhibition of biofilm formation was determined after crystal violet stain at its sub-inhibitory concentrations [45]. Briefly, serial dilutions of VFs were added to a 96-well plate (Orange Scientific, Braine-l’Alleud, Belgium) and incubated with different *Candida* spp. inoculums. The media with inoculums were used as positive controls of growth, and the Amphotericin B was used as drug standard at a concentration of 0.2 mg/mL. The morphology of *Candida* spp. was monitored using an inverted microscope (Olympus CK2) at 40× magnification. The minimum biofilm inhibitory concentration was also determined as the minimum concentration of substance without fungal development [46].

### 3.7. Biofilm Sustained Inhibition (BSI)

The standard optical density of biofilm sustained inhibition assay of *C. althaeoides* L. root VFs followed the previously reported protocol for the 96-well format of the biofilm screening assay [47] with some modification. Volatile fractions at different concentrations (7, 3.5, 1.75, 0.87, 0.44, 0.22, 0.11 and 0.05 mg/mL) were added during the 90 min adherence and 24 h growth steps of the Sustained Inhibition Biofilm Assay. Simultaneously different concentrations of Amphotericin B were tested ranging from 4 × 10^−3^ mg/mL to 0.03 × 10^−3^ mg/mL as standard drugs. The MIC concentrations for our fungi strains were run many times but not for this plate, if it is possible to mention it as follows. In brief, inoculums were adjusted to 2 × 10^6^ CFU mL^−1^ from overnight *Candida* spp. cultures. One hundred microliters of suspension were added to the 96-well plates and shaken at 37 °C for 90 min at 350 rpm in an APELEX shaker. Media was then removed, wells were washed with PBS, and fresh media (or media with VFs) was added back to wells. Plates were then resealed and shaken for a further 24 h at 37 °C. Finally, media was removed, and the absorbance (OD) at 570 nm was determined on a Multiskan™ FC Microplate Photometer.

### 3.8. Statistical Analysis

Results were reported as means ± standard deviation. All assays were performed considering three analytical replications. Based on the replications, one-way analysis of variance (ANOVA, followed by Turkey’s multiple range test at *p* < 0.05 level) was employed to assess the significant difference among the biological activities of the samples.

All statistical tests were performed by GraphPad Prism 6 software (GraphPad Software, Inc., San Diego, CA, USA). Heatmaps graphical representations were guided on OD of microdilution 96 plates at 570 nm.

## 4. Conclusions

The present research investigates for the first time the chemical profiles of the two VFs of *C. althaeoides* L. roots and their biological activities. A large number of volatile chemical compounds in the two VFs were identified reaching a value as high as 86 in the chloroformic extarct. These two VFs showed a good antifungal activity against three strains of *Candida* genus which are pathogenic years. Anti-biofilm activity in *Candida* spp. with up to 90% was IBF/BSI, indicating that they are promising sources of natural antifungal agents. The richness of the biologically active compounds in these two VFs may be responsible for this activity. Therefore, on the basis of the cytotoxicity study of these two VFs, such a species might be suitable for pharmaceutical applications.

## Figures and Tables

**Table 1:** List of major volatile compounds detected in the hexane fraction (VF1) of *C. althaeoides* L. roots by GC-MS.

N. Peak	Compound	Area%	LRI (Lib)	LRI (Exp)	Library	Compound Class	Formula
15	Myrtenol	0.93	1202	1198	FFNSC 4.0	Oxygenated monoterpene	C_10_H_16_O
17	Cuminaldehyde	0.79	1243	1245	FFNSC 4.0	Oxygenated monoterpene	C_10_H_12_O
18	Carvone	1.77	1246	1246	FFNSC 4.0	Oxygenated monoterpene	C_10_H_14_O
20	4-Vinylguaiacol	12.2	1309	1315	FFNSC 4.0	Alcohol	C_9_H_10_O_2_
21	Methyl 4-formylbenzoate	1.03	-	1370	W11N17	Ester	C_9_H_8_O_3_
24	Tetradec-1-ene	0.9	1379	1391	FFNSC 4.0	Alkene	C_14_H_28_
26	α-Methoxynaphthalene	1.52	1450	1453	FFNSC 4.0	Alkene	C_11_H_10_O
28	2,4-bis(1,1-dimethylethyl)-phenol	1.47	-	1509	W11N17	Alcohol	C_14_H_22_O
31	*n*-Hexadecene	1.1	1593	1592	FFNSC 4.0	Alkene	C_16_H_32_
39	Octadec-1-ene	1.03	1793	1792	FFNSC 4.0	Alkene	C_18_H_36_
42	diisobutyl phthalate	1.55	1858	1860	FFNSC 4.0	Ester	C_16_H_22_O_4_
48	*n*-Hexadecanoic acid	29.77	1977	1983	FFNSC 4.0	Fatty acid	C_16_H_32_O_2_
49	*n*-Eicosene	3.98	1994	1993	FFNSC 4.0	Alkene	C_20_H_40_
50	*n*-Eicosane	2.11	2000	1999	FFNSC 4.0	Alkane	C_20_H_42_
51	*n*-Octadecanol	2.89	2081	2087	FFNSC 4.0	Alcohol	C_18_H_38_O
53	Ethyl linoleate	1.75	2164	2160	FFNSC 4.0	Fatty acid	C_20_H_36_O_2_
54	Ethyl linolenate	1.47	2165	2166	FFNSC 4.0	Fatty acid	C_20_H_34_O_2_
56	*n*-Tricosane	0.81	2300	2299	FFNSC 4.0	Alkane	C_23_H_48_
57	Bis(2-ethylhexyl)-adipate	9.69	2392	2391	FFNSC 4.0	Ester	C_22_H_42_O_4_
58	*n*-Tetracosane	1.23	2400	2399	FFNSC 4.0	Alkane	C_24_H_50_
59	*n*-Pentacosane	1.59	2500	2499	FFNSC 4.0	Alkane	C_25_H_52_
62	*n*-Hexacosane	1.77	2600	2598	FFNSC 4.0	Alkane	C_26_H_54_
64	*n*-Heptacosane	1.64	2700	2699	FFNSC 4.0	Alkane	C_27_H_56_
66	*n*-Octacosane	1.37	2800	2798	FFNSC 4.0	Alkane	C_28_H_58_
68	*n*-Nonacosane	1.12	900	2898	FFNSC 4.0	Alkane	C_29_H_60_
73	3,5-bis(1,1-dimethylthyl)-4-hydroxy-, octadecyl ester	2.71	-	3596	W11N17	Carboxylic acid	C_35_H_62_O_3_

FFNSC: Flavor and Fragrance Natural and Synthetic Compounds; LRI: Linear Retention Indices; W11N17: Wiley11-Nist17.

**Table 2:** List of major volatile compounds detected in the chloroform fraction (VF2) of *C. althaeoides* L. roots by GC-MS.

N. Peak	Compound	Area%	LRI (Lib)	LRI (Exp)	Library	Compound Class	Formula
3	Hexan-2-ol	0.91	802	812	FFNSC 4.0	Alcohol	C_6_H_14_O
4	Furfural	3.24	845	832	FFNSC 4.0	Aldehyde	C_5_H_4_O_2_
9	*β*-Lutidine	1.29	955	955	FFNSC 4.0	*Pyridine*	C_7_H_9_N
12	3-ethenyl- Pyridine	2.43	-	966	W11N17	*Pyridine*	C_7_H_7_N
15	*n*-Hexanoic acid	1.91	997	1004	FFNSC 4.0	Carboxylic acid	C_6_H_12_O_2_
17	Benzyl alcohol	7.86	1040	1037	FFNSC 4.0	Alcohol	C_7_H_8_O
23	2-Methoxyphenol	1.27	-	1088	W11N17	Alcohol	C_7_H_8_O_2_
27	Phenethyl alcohol	2.07	1113	1115	FFNSC 4.0	Alcohol	C_8_H_10_O
35	4-Vinylphenol	0.98	1217	1223	FFNSC 4.0	Alcohol	C_8_H_8_O
41	Benzopyridine	1	1259	1263	FFNSC 4.0	Pyridine	C_9_H_7_N
43	4-Vinylguaiacol	2.87	1309	1314	FFNSC 4.0	Alcohol	C_7_H_8_O_2_
47	Vanillin	1.49	1394	1399	FFNSC 4.0	Aldehyde	C_8_H_8_O_3_
66	*n*-Hexadecanoic acid	34.01	1977	1983	FFNSC 4.0	Fatty acid	C_16_H_32_O_2_
67	*n*-Octadecanol	3.53	2081	2087	FFNSC 4.0	Alcohol	C_18_H_38_O
69	Linoleic acid	7.3	2144	2154	FFNSC 4.0	Fatty acid	C_18_H_32_O_2_
70	*n*-Docosane	2.43	2200	2199	FFNSC 4.0	Alkane	C_22_H_46_
72	Bis(2-ethylhexyl)-adipate	3.57	2392	2390	FFNSC 4.0	Ester	C_22_H_42_O_4_
74	*n*-Pentacosane	1.47	2500	2499	FFNSC 4.0	Alkane	C_25_H_52_
86	3,5-bis(1,1-dimethylthyl)-4-hydroxy-, octadecyl ester	0.95	-	3595	W11N17	Carboxylic acid	C_35_H_62_O_3_

FFNSC: Flavor and Fragrance Natural and Synthetic Compounds; LRI: Linear Retention Indices; W11N17: Wiley11-Nist17.

## Data Availability

Not applicable.

## Supplementary Materials

The following supporting information can be downloaded at: https://www.mdpi.com/article/10.3390/molecules27206834/s1, Table S1: List of volatile compounds detected in the hexane fraction (VF1) of *C. althaeoides* L. roots by GC-MS; Table S2: List of volatile compounds detected in the chloroform fraction (VF2) of *C. althaeoides* L. roots by GC-MS.

## Sample Availability

Samples of the compounds are available from the authors.
