# Supplementary material for: Chemical Composition, Antifungal and Anti-Biofilm Activities of Volatile Fractions of Convolvulus althaeoides L. Roots from Tunisia
Source: Molecules. 2022 Oct 12;27(20):6834. doi: 10.3390/molecules27206834 (PMC9609869; doi:10.3390/molecules27206834)
Supplement: Supplementary file 1 [file molecules-27-06834-s001.zip › molecules-1935668 supplementary.pdf]

molecules

MDPI

Article

Chemical Composition, Antifungal and Anti-Biofilm Activities of Volatile Fractions of *Convolvulus althaeoides* L. Roots from Tunisia

Soukaina Hrichi [1], Raja Chaâbane-Banaoues [2], Filippo Alibrando [3], Ammar B. Altemimi [4,5], Oussama Babba [2], Yassine Oulad El Majdoub [6], Habib Nasri [1], Luigi Mondello [3,6,7], Hamouda Babba [2], Zine Mighri [1] and Francesco Cacciola [8,*]

[1] Laboratory of Physico-Chemistry of Materials, Faculty of Sciences of Monastir, University of Monastir, Monastir 5000, Tunisia; soukaina.hrichi@gmail.com (S.H.); hnasri1@gmail.com (H.N.); zinemighri@yahoo.fr (Z.M.)
[2] Laboratory of Parasitology and Mycology (LP3M), Department of Clinical Biology, Faculty of Pharmacy of Monastir, University of Monastir, Monastir 5000, Tunisia; rajachaabanebanaoues@gmail.com (R.C.-B.); ouba90@gmail.com (O.B.); hamouda.babba@gnet.tn (H.B.)
[3] Chromaleont s.r.l., c/o Department of Chemical, Biological, Pharmaceutical and Environmental Sciences, University of Messina, 98122 Messina, Italy; filippo.alibrando@chromaleont.it (F.A.); lmondello@unime.it (L.M.)
[4] Department of Food Science, College of Agriculture, University of Basrah, Basraq 61004, Iraq; ammar.ramddan@uobasrah.edu.iq
[5] College of Medicine, University of Warith Al-Anbiyaa, Karbala 56001, Iraq
[6] Department of Chemical, Biological, Pharmaceutical and Environmental Sciences, University of Messina, 98122 Messina, Italy; yassine.ouladelmajdoub@unime.it
[7] Department of Sciences and Technologies for Human and Environment, University Campus Bio-Medico of Rome, 00128 Rome, Italy
[8] Department of Biomedical, Dental, Morphological and Functional Imaging Sciences, University of Messina, 98122 Messina, Italy
* Correspondence: cacciolaf@unime.it

Table S1. List of volatile compounds detected in the hexane fraction (VF1) of *C. althaeoides* L. roots by GC-MS.

N. Peak	Compound	Area%	LRI (lib)	LRI (exp)	Library	Compound Class	Formula
1	Hexan-3-ol	0.03	795	799	FFNSC 4.0	Alcohol	$C_6H_{14}O$
2	*n*-Hexanal	0.07	801	802	FFNSC 4.0	Aldehyde	$C_6H_{12}O$
3	Benzaldehyde	0.07	960	963	FFNSC 4.0	Aldehyde	C_7H_6O
4	1-Octen-3-ol	0.21	978	979	FFNSC 4.0	Alcohol	$C_8H_{16}O$
5	*n*-Octanal	0.02	1006	1004	FFNSC 4.0	Aldehyde	$C_8H_{16}O$
6	2-Ethylhexanol	0.17	1030	1029	FFNSC 4.0	Alcohol	$C_8H_{18}O$
7	Benzyl alcohol	0.17	1040	1037	FFNSC 4.0	Alcohol	C_7H_8O
8	(*E*)-Linalool oxide	0.06	1086	1087	FFNSC 4.0	Oxygenated monoterpene	$C_{10}H_{18}O_2$
9	Guaiacol	0.05	1104	1089	FFNSC 4.0	Alcohol	$C_7H_8O_2$
10	Linalyl anthranilate	0.23	1104	1101	FFNSC 4.0	Ester	$C_{17}H_{23}NO_2$
11	*n*-Nonanal	0.14	1107	1105	FFNSC 4.0	Aldehyde	$C_9H_{18}O$
12	Phenethyl alcohol	0.11	1113	1115	FFNSC 4.0	Alcohol	$C_8H_{10}O$
13	Dodec-1-ene	0.2	1191	1191	FFNSC 4.0	Alkene	$C_{12}H_{24}$
14	α-Terpineol	0.17	1195	1197	FFNSC 4.0	Oxygenated monoterpene	$C_{10}H_{18}O$
15	Myrtenol	0.93	1202	1198	FFNSC 4.0	Oxygenated monoterpene	$C_{10}H_{16}O$
16	*n*-Decanal	0.12	1208	1207	FFNSC 4.0	Aldehyde	$C_{10}H_{20}O$

17	Cuminaldehyde	0.79	1243	1245	FFNSC 4.0	Oxygenated monoterpene	$C_{10}H_{12}O$
18	Carvone	1.77	1246	1246	FFNSC 4.0	Oxygenated monoterpene	$C_{10}H_{14}O$
19	Geraniol	0.13	1255	1253	FFNSC 4.0	Oxygenated monoterpene	$C_{10}H_{18}O$
20	4-Vinylguaiacol	12.2	1309	1315	FFNSC 4.0	Alcohol	$C_9H_{10}O_2$
21	Methyl 4-formylbenzoate	1.03	-	1370	W11N17	Ester	$C_9H_8O_3$
22	Butyl benzoate	0.19	1376	1375	FFNSC 4.0	Ester	$C_{11}H_{14}O_2$
23	(*E*), β-Damascenone	0.13	1379	1380	FFNSC 4.0	Apocarotene	$C_{13}H_{18}O$
24	Tetradec-1-ene	0.9	1379	1391	FFNSC 4.0	Alkene	$C_{14}H_{28}$
25	*n*-Tetradecane	0.32	1400	1400	FFNSC 4.0	Alkane	$C_{14}H_{30}$
26	*α*-Methoxynaphthalene	1.52	1450	1453	FFNSC 4.0	Alkene	$C_{11}H_{10}O$
27	*n*-Pentadecane	0.22	1500	1499	FFNSC 4.0	Alkane	$C_{15}H_{32}$
28	2,4-Bis(1,1-dimethylethyl)-phenol	1.47	-	1509	W11N17	Alcohol	$C_{14}H_{22}O$
29	3-Nonyl-tiglate	0.27	1504	1514	FFNSC 4.0	Ester	$C_{14}H_{26}O_2$
30	2-[[[4-(4-hydroxy-4-methylpentyl)-, 3-cyclohexen-1-yl]methylene]amino]-, Methyl-benzoate	0.89	1589	1590	FFNSC 4.0	Ester	$C_{21}H_{29}NO_3$
31	*n*-Hexadecene	1.1	1593	1592	FFNSC 4.0	Alcene	$C_{16}H_{32}$
32	*n*-Hexadecane	0.21	1600	1599	FFNSC 4.0	Alkane	$C_{16}H_{34}$
33	Benzophenone	0.32	1627	1631	FFNSC 4.0	Apocarotene	$C_{13}H_{10}O$
34	Hedione	0.19	1650	1652	FFNSC 4.0	Apocarotene	$C_{13}H_{22}O_3$
35	Cadalene	0.11	1677	1676	FFNSC 4.0	Sesquiterpene hydrocarbon	$C_{15}H_{18}$
36	*n*-Heptadecane	0.13	1700	1699	FFNSC 4.0	Alkane	$C_{17}H_{36}$
37	Pentadecanal	0.15	-	1715	W11N17	Aldehyde	$C_{15}H_{30}O$
38	*n*-Pentadecanol	0.17	1782	1786	FFNSC 4.0	Alcohol	$C_{15}H_{32}O$
39	Octadec-1-ene	1.03	1793	1792	FFNSC 4.0	Alkene	$C_{18}H_{36}$
40	*n*-Octadecane	0.27	1800	1799	FFNSC 4.0	Alkane	$C_{18}H_{38}$
41	Phytone	0.32	1841	1841	FFNSC 4.0	Ketone	$C_{18}H_{36}O$
42	diisobutyl phthalate	1.55	1858	1860	FFNSC 4.0	Ester	$C_{16}H_{22}O_4$
43	Pentadecylic acid	0.42	1869	1870	FFNSC 4.0	Oxygenated sesquiterpene	$C_{15}H_{30}O_2$
44	*n*-Hexadecanol	0.36	1884	1884	FFNSC 4.0	Alcohol	$C_{16}H_{34}O$
45	*n*-Nonadecane	0.28	1900	1899	FFNSC 4.0	Alkane	$C_{19}H_{40}$
46	7,9-Di-tert-butyl-1-oxaspiro(4,5)deca-6,9-diene-2,8-dione	0.65	-	1905	W11N17	Apocarotene	$C_{17}H_{24}O_3$
47	Methyl hexadecanoate	0.51	1925	1925	FFNSC 4.0	Fatty acid	$C_{17}H_{34}O_2$
48	*n*-Hexadecanoic acid	29.77	1977	1983	FFNSC 4.0	Fatty acid	$C_{16}H_{32}O_2$
49	*n*-Eicosene	3.98	1994	1993	FFNSC 4.0	Alkene	$C_{20}H_{40}$
50	*n*-Eicosane	2.11	2000	1999	FFNSC 4.0	Alkane	$C_{20}H_{42}$
51	*n*-Octadecanol	2.89	2081	2087	FFNSC 4.0	Alcohol	$C_{18}H_{38}O$
52	5-dodecyldihydro-2(3*H*)-furanone	0.52	-	2104	W11N17	Ketone	$C_{16}H_{30}O_2$
53	Ethyl linoleate	1.75	2164	2160	FFNSC 4.0	Fatty acid	$C_{20}H_{36}O_2$
54	Ethyl linolenate	1.47	2165	2166	FFNSC 4.0	Fatty acid	$C_{20}H_{34}O_2$

55	*n*-Docosane	0.4	2200	2199	FFNSC 4.0	Alkane	$C_{22}H_{46}$
56	*n*-Tricosane	0.81	2300	2299	FFNSC 4.0	Alkane	$C_{23}H_{48}$
57	Bis(2-ethylhexyl)-adipate	9.69	2392	2391	FFNSC 4.0	Ester	$C_{22}H_{42}O_4$
58	*n*-Tetracosane	1.23	2400	2399	FFNSC 4.0	Alkane	$C_{24}H_{50}$
59	*n*-Pentacosane	1.59	2500	2499	FFNSC 4.0	Alkane	$C_{25}H_{52}$
60	Bis(2-ethylhexyl)-phthalate	0.69	2531	2532	FFNSC 4.0	Ester	$C_{24}H_{38}O_4$
61	2-Methylpentacosane	0.11	-	2561	W11N17	Alkane	$C_{26}H_{54}$
62	*n*-Hexacosane	1.77	2600	2598	FFNSC 4.0	Alkane	$C_{26}H_{54}$
63	2-Methylhexacosane	0.1	-	2671	W11N17	Alkane	$C_{27}H_{56}$
64	*n*-Heptacosane	1.64	2700	2699	FFNSC 4.0	Alkane	$C_{27}H_{56}$
65	2-Methylheptacosane	0.06	-	2771	W11N17	Alkane	$C_{28}H_{58}$
66	*n*-Octacosane	1.37	2800	2798	FFNSC 4.0	Alkane	$C_{28}H_{58}$
67	Squalene	0.28	2810	2811	FFNSC 4.0	Triterpene	$C_{30}H_{50}$
68	*n*-Nonacosane	1.12	900	2898	FFNSC 4.0	Alkane	$C_{29}H_{60}$
69	*n*-Triacontane	0.69	3000	2998	FFNSC 4.0	Alkane	$C_{30}H_{62}$
70	*n*-Hentriacontane	0.47	3100	3098	FFNSC 4.0	Alkane	$C_{31}H_{64}$
71	*n*-Dotriacontane	0.31	3200	3199	FFNSC 4.0	Alkane	$C_{32}H_{66}$
72	*n*-Tritriacontane	0.12	3300	3298	FFNSC 4.0	Alkane	$C_{33}H_{68}$
73	3,5-bis(1,1-dimethylthyl)-4-hydroxy-, octadecyl ester	2.71	-	3596	W11N17	Carboxylic acid	$C_{35}H_{62}O_3$

FFNSC: Flavor and Fragrance Natural and Synthetic Compounds; LRI: Linear Retention Indices; W11N17: Wiley11-Nist17.

Table S2. List of volatile compounds detected in the chloroform fraction (VF2) of *C. althaeoides* L. roots by GC-MS.

N. Peak	Compound	Area%	LRI (lib)	LRI (exp)	Library	Compound Class	Formula
1	1-Methyl- cyclopentanol	0.07	-	798	W11N17	Alcohol	$C_6H_{12}O$
2	Hexan-3-ol	0.07	795	799	FFNSC 4.0	Alcohol	$C_6H_{14}O$
3	Hexan-2-ol	0.91	802	812	FFNSC 4.0	Alcohol	$C_6H_{14}O$
4	Furfural	3.24	845	832	FFNSC 4.0	Aldehyde	$C_5H_4O_2$
5	Furfuryl alcohol	0.3	849	852	FFNSC 4.0	Alcohol	$C_5H_6O_2$
6	Hex-(3Z)-enol	0.37	853	852	FFNSC 4.0	Alcohol	$C_6H_{12}O$
7	*n*-Hexanol	0.23	867	866	FFNSC 4.0	Alcohol	$C_6H_{14}O$
8	2-acetyl-furan	0.1	913	911	FFNSC 4.0	Ketone	$C_6H_5ClO_2$
9	β-Lutidine	1.29	955	955	FFNSC 4.0	*Pyridine*	C_7H_9N
10	5-Methyl furfural	0.21	960	961	FFNSC 4.0	Aldehyde	$C_6H_6O_2$
11	Benzaldehyde	0.75	960	963	FFNSC 4.0	Aldehyde	C_7H_6O
12	3-Ethenylpyridine	2.43	-	966	W11N17	*Pyridine*	C_7H_7N
13	1-Octen-3-ol	0.1	978	980	FFNSC 4.0	Alcohol	$C_8H_{16}O$
14	2,4,6-Trimethylpyridine	0.16	986	990	FFNSC 4.0	*Pyridine*	$C_8H_{11}N$
15	*n*-Hexanoic acid	1.91	997	1004	FFNSC 4.0	Carboxylic acid	$C_6H_{12}O_2$
16	2-Ethyl-hexanol	0.07	1030	1029	FFNSC 4.0	Hexanol	$C_6H_{14}O_2$
17	Benzyl alcohol	7.86	1040	1037	FFNSC 4.0	Alcohol	C_7H_8O
18	Phenylacetaldehyde	0.1	1045	1045	FFNSC 4.0	Aldehyde	C_8H_8O
19	γ-Hexalactone	0.09	1060	1053	FFNSC 4.0	Ester	$C_6H_{10}O_2$
20	*o*-Cresol	0.11	1051	1055	FFNSC 4.0	Alcohol	C_7H_8O
21	Diethyl malonate	0.19	1071	1068	FFNSC 4.0	Oxygenated monoterpene	$C_{10}H_{16}O_5$
22	(*Z*)-Linalool oxide	0.3	1069	1071	FFNSC 4.0	Oxygenated monoterpene	$C_{10}H_{18}O_2$

23	2-Methoxyphenol	1.27	-	1088	W11N17	Alcohol	$C_7H_8O_2$
24	Linalyl anthranilate	0.16	1104	1101	FFNSC 4.0	Ester	$C_{17}H_{23}NO_2$
25	*n*-Nonanal	0.22	1107	1106	FFNSC 4.0	Aldehyde	$C_9H_{18}O$
26	1-(3-Pyridinyl)-ethanone	0.07	-	1112	W11N17	*Pyridine*	C_7H_7NO
27	Phenethyl alcohol	2.07	1113	1115	FFNSC 4.0	Alcohol	$C_8H_{10}O$
28	Methyl nicotinate	0.81	1142	1140	FFNSC 4.0	Ester	$C_7H_7NO_2$
29	Oxophorone	0.07	1148	1146	FFNSC 4.0	Ketone	$C_9H_{12}O_2$
30	Benzyl acetate	0.06	-	1164	W11N17	Ester	C9H10O2
31	Dodec-1-ene	0.12	1191	1191	FFNSC 4.0	Alkene	$C_{12}H_{24}$
32	α-Terpineol	0.07	1195	1197	FFNSC 4.0	Oxygenated monoter-pene	$C_{10}H_{18}O$
33	Myrtenol	0.46	1202	1199	FFNSC 4.0	Oxygenated monoter-pene	$C_{10}H_{16}O$
34	*n*-Decanal	0.21	1208	1207	FFNSC 4.0	Aldehyde	$C_{10}H_{20}O$
35	4-Vinylphenol	0.98	1217	1223	FFNSC 4.0	Alcohol	C_8H_8O
36	Benzosulfonazole	0.08	1226	1227	FFNSC 4.0	-	C_7H_5NS
37	1-Azanaphthalene	0.11	1237	1240	FFNSC 4.0	Pyridine	C_9H_7N
38	Cuminaldehyde	0.23	1243	1244	FFNSC 4.0	Oxygenated monoter-pene	$C_{10}H_{12}O$
39	Carvone	0.68	1246	1246	FFNSC 4.0	Oxygenated monoter-pene	$C_{10}H_{14}O$
40	*p*-Anisaldehyde	0.14	1257	1258	FFNSC 4.0	Aldehyde	$C_8H_8O_2$
41	Benzopyridine	1	1259	1263	FFNSC 4.0	Pyridine	C_9H_7N
42	*p*-Cymen-7-ol	0.23	1291	1295	FFNSC 4.0	Oxygenated monoter-pene	$C_{10}H_{14}O$
43	4-Vinylguaiacol	2.87	1309	1314	FFNSC 4.0	Alcohol	$C_7H_8O_2$
44	γ-Nonalactone	0.18	1362	1362	FFNSC 4.0	Ester	$C_9H_{16}O_2$
45	Methyl 4-formylbenzoate	0.76	-	1370	W11N17	Ester	$C_9H_8O_3$
46	Tetradec-1-ene	0.25	1392	1391	FFNSC 4.0	Alkene	$C_{14}H_{28}$
47	Vanillin	1.49	1394	1399	FFNSC 4.0	Aldehyde	$C_8H_8O_3$
48	bis-1,1′-(1,3-phenylene) Ethanone	0.06	-	1434	W11N17	Ketone	$C_{14}H_{12}O$
49	5,6,7,7a-Tetrahydro-4,4,7a-trimethyl-2(4*H*)-benzofuranone	0.07	-	1530	W11N17	Apocarotene	$C_{11}H_{16}O_2$
50	2-[[[4-(4-hydroxy-4-methylpentyl)-, 3-cyclo-hexen-1-yl]meth-ylene]amino]-, Methyl-benzoate	0.27	1589	1589	FFNSC 4.0	Ester	$C_{21}H_{29}NO_3$
51	*n*-Hexadecene	0.51	1593	1592	FFNSC 4.0	Alkene	$C_{16}H_{32}$
52	*n*-Hexadecane	0.17	1600	1599	FFNSC 4.0	Alkane	$C_{16}H_{34}$
53	5,7-diepi-alpha-eudesmol	0.1	1610	1608	FFNSC 4.0	Oxygenated sesquiter-pene	$C_{15}H_{26}O$
54	*n*-Tetradecanal	0.08	1614	1614	FFNSC 4.0	Alkane	$C_{14}H_{28}O$
55	Benzophenone	0.1	1627	1631	FFNSC 4.0	Apocarotene	$C_{13}H_{10}O$
56	1,6-dimethyl-4-(1-meth-ylethyl)-naphthalene	0.09	-	1675	W11N17	Sesquiterpene hydro-carbon	$C_{15}H_{18}$
57	*n*-Pentadecanal	0.15	-	1716	W11N17	Aldehyde	$C_{15}H_{30}O$
58	(E)-Coniferyl alcohol	0.64	1732	1741	FFNSC 4.0	Phenylpropanoid	$C_{10}H_{12}O_3$

59	Octadec-1-ene	0.67	1793	1792	FFNSC 4.0	Alkene	$C_{18}H_{36}$
60	*n*-Octadecane	0.16	1800	1799	FFNSC 4.0	Alkane	$C_{18}H_{38}$
61	Phytone	0.36	1841	1841	FFNSC 4.0	Ketone	$C_{18}H_{36}O$
62	diisobutyl phthalate	0.63	1858	1860	FFNSC 4.0	Ester	$C_{16}H_{22}O_4$
63	Pentadecylic acid	0.36	1869	1870	FFNSC 4.0	Oxygenated sesquiterpene	$C_{15}H_{30}O_2$
64	*n*-Hexadecanol	0.2	1884	1883	FFNSC 4.0	Alcohol	$C_{16}H_{34}O$
65	Methyl hexadecanoate	0.43	1925	1936	FFNSC 4.0	Fatty acid	$C_{17}H_{34}O_2$
66	*n*-Hexadecanoic acid	34.01	1977	1983	FFNSC 4.0	Fatty acid	$C_{16}H_{32}O_2$
67	*n*-Octadecanol	3.53	2081	2087	FFNSC 4.0	Alcohol	$C_{18}H_{38}O$
68	5-Dodecyldihydro-2(3*H*)-furanone	0.58	-	2104	W11N17	Ketone	$C_{16}H_{30}O_2$
69	Linoleic acid	7.3	2144	2154	FFNSC 4.0	Fatty acid	$C_{18}H_{32}O_2$
70	*n*-Docosane	2.43	2200	2199	FFNSC 4.0	Alkane	$C_{22}H_{46}$
71	*n*-Tricosane	0.52	2300	2299	FFNSC 4.0	Alkane	$C_{23}H_{48}$
72	Bis(2-ethylhexyl)-adipate	3.57	2392	2390	FFNSC 4.0	Ester	$C_{22}H_{42}O_4$
73	*n*-Tetracosane	0.35	2400	2399	FFNSC 4.0	Alkane	$C_{24}H_{50}$
74	*n*-Pentacosane	1.47	2500	2499	FFNSC 4.0	Alkane	$C_{25}H_{52}$
75	Bis(2-ethylhexyl)-phthalate	0.79	2531	2532	FFNSC 4.0	Ester	$C_{24}H_{38}O_4$
76	Benzyl hexadecenoate	0.33	-	2578	W11N17	Ester	$_{23}H_{38}O_2$
77	*n*-Hexacosane	0.43	2600	2598	FFNSC 4.0	Alkane	$C_{26}H_{54}$
78	*n*-Heptacosane	0.78	2700	2699	FFNSC 4.0	Alkane	$C_{27}H_{56}$
79	*n*-Octacosane	0.61	2800	2799	FFNSC 4.0	Alkane	$C_{28}H_{58}$
80	Squalene	0.34	2810	2811	FFNSC 4.0	Triterpene	$C_{30}H_{50}$
81	*n*-Nonacosane	0.56	2900	2899	FFNSC 4.0	Alkane	$C_{29}H_{60}$
82	*n*-Triacontane	0.34	3000	2998	FFNSC 4.0	Alkane	$C_{30}H_{62}$
83	*n*-Hentriacontane	0.3	3100	3098	FFNSC 4.0	Alkane	$C_{31}H_{64}$
84	*n*-Dotriacontane	0.18	3200	3199	FFNSC 4.0	Alkane	$C_{32}H_{66}$
85	*n*-Tritriacontane	0.13	3300	3299	FFNSC 4.0	Alkane	$C_{33}H_{68}$
86	3,5-bis(1,1-dimethylthyl)-4-hydroxy-, octadecyl ester	0.95	-	3595	W11N17	Carboxylic acid	$C_{35}H_{62}O_3$

FFNSC: Flavor and Fragrance Natural and Synthetic Compounds; LRI: Linear Retention Indices; W11N17: Wiley11-Nist17.
